# Effects of variable resistance training on lower limb explosive power in athletes: a systematic review and meta-analysis

**DOI:** 10.7717/peerj.20644

**Published:** 2026-02-04

**Authors:** Ziqi Xu, Songpeng Su, Zitong Xu

**Affiliations:** 1 Department of Physical Education, Sun Yat-sen University, Guangzhou, China; 2 School of Athletic Training, Guangzhou Sport University, Guangzhou, China; 3 School of Physical Education, Wuhan University of Technology, Wuhan, China

**Keywords:** Elastic band training, Chain training, Resistance training, Athletes, Lower limb explosive power

## Abstract

**Objectives:**

This study aims to investigate the effects of variable resistance training (VRT) on athletes’ lower limb explosive power through assessments of jumping, sprinting, and change of direction (COD) performance.

**Methods:**

A systematic search was conducted across four electronic databases (Web of Science, PubMed, Scopus, ProQuest) from their inception until March 23, 2025. Study eligibility was assessed against the Population, Intervention, Comparison, Outcomes, Study design (PICOS) framework. Following data extraction, the methodological quality of the included studies was assessed using the Cochrane Risk of Bias tool. Data analysis was performed using Stata 15 software, with standardized mean differences (SMD) and 95% confidence intervals calculated as the pooled effect size measure.

**Results:**

Fifteen articles involving 442 participants were included in the final analysis. The overall meta-analysis demonstrated that VRT was an effective method for enhancing athletes’ jumping performance (SMD = 0.81 cm, *p* < 0.001), sprint performance (SMD = −1.13 s, *p* < 0.001), and COD performance (SMD = −1.66 s, *p* < 0.001). Subgroup analyses indicated significant positive effects of VRT on vertical jump (VJ: SMD = 0.31 cm, *p* = 0.027), squat jump (SJ: SMD = 0.94 cm, *p* < 0.001), countermovement jump (CMJ: SMD = 1.01 cm, *p* < 0.001), 5-m sprint (SMD = −1.18 s, *p* < 0.001), 10-m sprint (SMD = −1.18 s, *p* < 0.001), 30-m sprint (SMD = −1.08 s, *p* = 0.013), T-test (SMD = −2.01 s, *p* < 0.001), repeated change of direction (RCOD: SMD = −2.01 s, *p* < 0.001), and Illinois test (SMD = −1.85 s, *p* < 0.001).

**Conclusion:**

This systematic review suggests that VRT may serve as an effective training strategy for enhancing lower-limb explosive power in athletes. However, due to significant heterogeneity among the included studies and potential publication bias, the definitive benefits of VRT require further validation through additional high-quality research.

## Introduction

Lower limb explosive power is essential for athletic performance. It plays a key role in sports that involve jumping, sprinting, and rapid changes of direction. It is defined as the ability of the neuromuscular system to generate power in an extremely brief period (Sawyer et al., 2021; Suchomel, Nimphius & Stone, 2016). This capacity directly influences movement execution and competitive outcomes in activities such as basketball jump shots (Erculj, Blas & Bracic, 2010), football breakthroughs (Beato et al., 2018), volleyball spikes, and sprinting (Vanderka et al., 2016). Numerous studies have consistently shown that elite athletes exhibit significantly higher levels of lower limb explosive power compared to average athletes, and this power is strongly positively correlated with athletic performance (Suchomel, Nimphius & Stone, 2016). Constant resistance training (CRT) has long been a prevalent method for developing explosive power and is widely applied in sports training. By utilizing high loads to stimulate the central nervous system, CRT enhances muscle fiber recruitment and contraction efficiency, enabling muscles to contract more powerfully and quickly. This, in turn, improves the rate of force development (RFD) and enhances explosive power performance (Cormie, McGuigan & Newton, 2011). The efficacy of CRT in increasing strength has been well-documented in numerous studies (Aagaard et al., 2002; Ataee et al., 2014).

However, CRT has notable limitations in practical application. The muscle strength required within the range of joint motion is positively correlated with the training effect (Galpin et al., 2015). However, due to mechanical disadvantages at specific joint angles, CRT often results in a mismatch between the torque generated by the weight and the torque produced by the muscles within the range of joint motion (Elliott, Wilson & Kerr, 1989; Frost, Cronin & Newton, 2010). Particularly when the movement reaches an individual’s weakest range of joint strength (the sticking point), the body must decelerate to overcome external resistance (Van den Tillaar, Andersen & Saeterbakken, 2014). This deceleration not only restricts muscle activation throughout the entire range of motion but may also negatively impact the development of explosive power (Haff & Triplett, 2016). Consequently, under CRT conditions, maximum muscle activation occurs only in the early stage of concentric movement and cannot be maximized throughout the entire range of motion (Shi et al., 2022).

To address these limitations, variable resistance training (VRT) has emerged as an innovative training method. By incorporating devices such as cams, elastic bands, or chains, VRT provides progressive resistance throughout the range of joint motion, achieving a training effect that approaches or reaches maximum strength output (Anderson, Sforzo & Sigg, 2008). This method not only effectively enhances maximum strength and explosive power but also offers the advantages of improving training safety and preventing sports injuries (Ghigiarelli et al., 2009). Studies have demonstrated that VRT significantly increases the activation of the quadriceps and hamstrings during the concentric contraction phase of the squat (Andersen et al., 2016). Moreover, the total resistance of VRT is similar to that of constant resistance in the pre-sticking region (98%), but it exhibits greater resistance characteristics in the sticking point (105%) and post-sticking region (113%) (Saeterbakken, Andersen & Van den Tillaar, 2016). These characteristics enable VRT to better simulate the strength demands of actual sports, thereby enhancing training effectiveness.

Previous research has explored the long-term effects of VRT on muscle hypertrophy (Fuentes-García, Malchrowicz-Mosko & Castañeda Babarro, 2024), maximum strength (Lin et al., 2022), and muscle mass (Tsai et al., 2022) through meta-analysis, as well as its acute effects on strength and speed (Shi et al., 2022). However, no meta-analysis has specifically targeted the long-term effects of VRT on lower limb explosive power. Despite numerous studies on the impact of VRT on athletes’ lower limb explosive power, the training effects of VRT on explosive power may vary due to different training periods, sports, and load parameters (Joy et al., 2016; Loturco et al., 2022). Therefore, this systematic review and meta-analysis aims to evaluate the effects of VRT on jumping, sprinting, and change-of-direction (COD) performance in athletes compared to CRT or control conditions. The findings will provide scientific evidence for implementing VRT in power training regimens.

## Information and Research Methods

This systematic review and meta-analysis strictly followed the Preferred Reporting Items for Systematic Reviews and Meta-Analyses (PRISMA) guidelines and was registered with PROSPERO (ID: CRD420251009742) (Moher et al., 2010) The outcomes reported in this paper are consistent with the PROSPERO-registered protocol. Both the original and any amended versions of the protocol are accessible on the PROSPERO website.

## Search strategy

**Figure 1:**
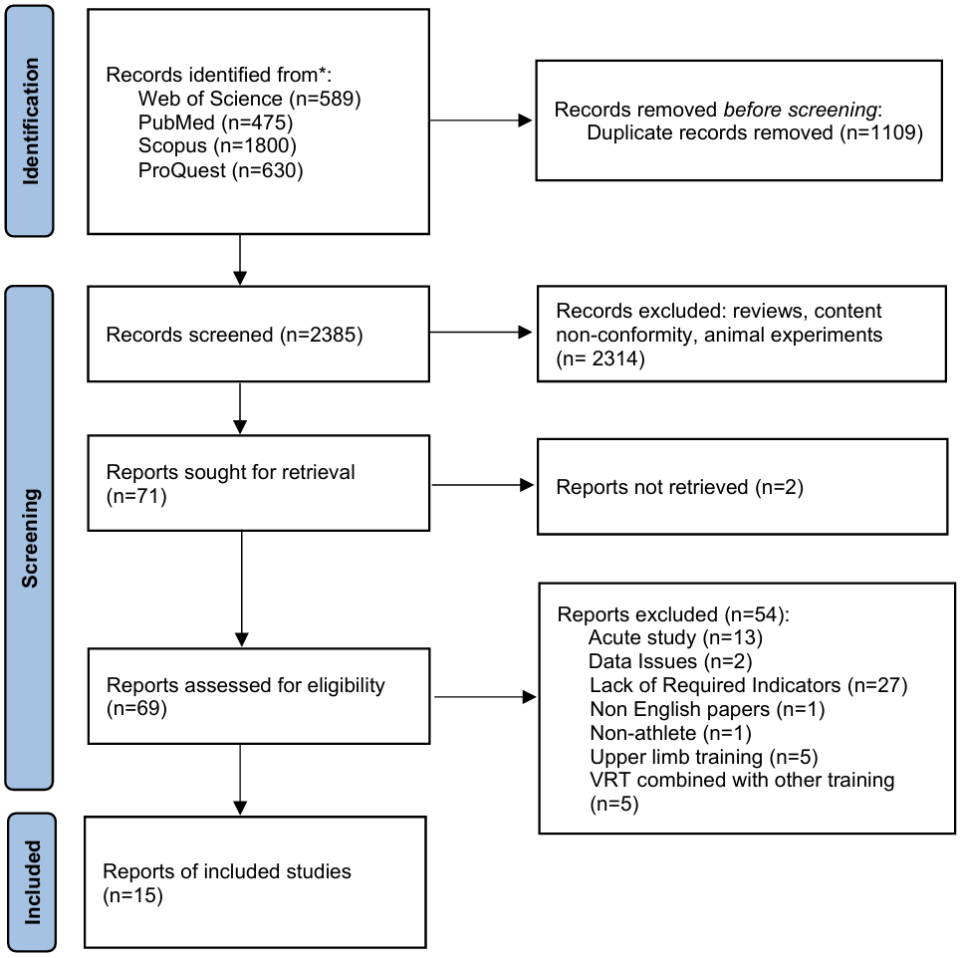
PRISMA flow chart for inclusion and exclusion of studies.

**Figure 2:**
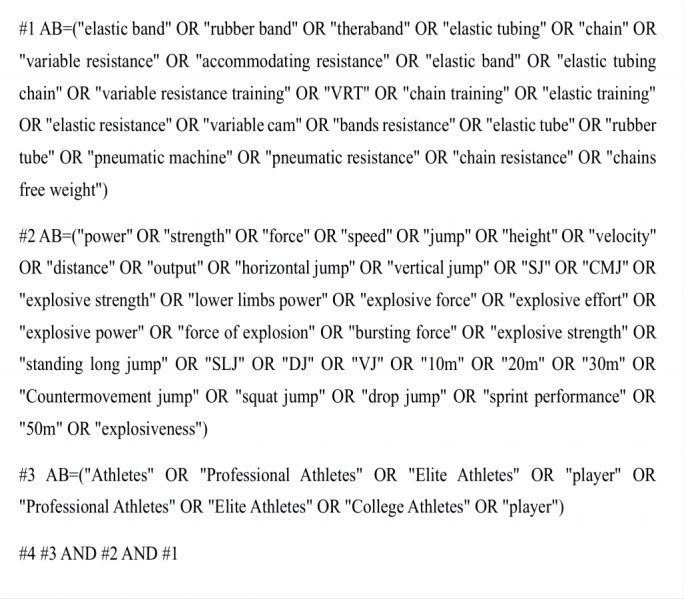
Web of Science literature selection strategy study selection.

The research was conducted in strict accordance with the PRISMA guidelines, and the detailed search process is shown in Fig. 1. The search for relevant literature was conducted across multiple databases, including PubMed (*n* = 475), Web of Science (*n* = 589), Scopus (*n* = 1,800), and ProQuest (*n* = 630). The search spanned from the inception of each database to March 23, 2025, with the language restricted to English. The search terms included elastic band, variable resistance training, VRT, chain training, elastic training, elastic resistance, chain resistance, power, speed, jump, change of direction, agility, COD, explosive strength, athletes, professional athletes, elite athletes, and so on. The Boolean operators “AND” and “OR” were used to combine search terms during the retrieval process. The search process is shown in Fig. 2 (taking Web of Science as an example). To ensure the accuracy and comprehensiveness of the search, two researchers (ZX and SS) independently reviewed and verified the search terms. In cases of disagreement, a third researcher (ZX) was consulted to make the final decision. The search strategy combined subject headings with free-text keywords, and when necessary, supplementary searches were conducted using the hand-searching method to identify additional relevant studies.

### Study selection

This study determined the inclusion criteria based on the Population, Intervention, Comparison, Outcomes, and Study design (PICOS) principle (Liberati et al., 2009).

The specific inclusion criteria were as follows: (1) The study subjects were athletes from various sports, with no injuries, illnesses, or other clinical symptoms. (2) The intervention for the experimental group was variable resistance training (chains/elastic bands), and apart from the intervention, the experimental group participated in the same sports training as the control group. (3) The control group underwent traditional resistance training or routine sport-specific training. (4) The outcome measures included Countermovement Jump (CMJ), Squat Jump (SJ), Vertical Jump (VJ), 5-m sprint, 10-m sprint, 30-m sprint, T test, repeated change of direction (RCOD) and illinois test, which reflect lower limb explosive power. (5) All included studies were randomized controlled trials (RCTs).

The exclusion criteria were as follows: (1) Reviews and conference papers. (2) Studies whose full text or research data could not be obtained. (3) Non-randomized controlled trials and self-controlled trials. (4) Participants who were not athletes or athletes with injuries or illnesses that could affect training.

A total of 15 articles met the requirements and were included in this study. The specific process of inclusion and exclusion is shown in Fig. 1.

### Data extraction

All retrieved literature was imported into EndNote X9 software for duplicate removal. The titles and abstracts of the remaining records were then screened for preliminary inclusion. After the studies to be included in the meta-analysis were finally determined, the data were extracted into a Microsoft Excel spreadsheet. The extracted data included: (1) The author’s name and the publication year. (2) The participants’ characteristics: gender, sport, age, and number of participants. (3) The training program: the intervention methods, duration, training frequency, outcome measures, and their mean values and standard deviations for both the experimental and control groups.

Pre- and pos*t*-test data for outcome measures were included as mean ±standard deviation. For the pooled analysis of pre-post differences, these data were converted into change scores. The standard deviation of the change score (SD_change) was calculated using the following formula:

\begin{eqnarray*}{\mathrm{SD}}_{\mathrm{change}}=\sqrt{{\mathrm{SD}}_{\mathrm{baseline}}^{2}+{\mathrm{SD}}_{\mathrm{post}}^{2}-2\times r\times {\mathrm{SD}}_{\mathrm{baseline}}\times {\mathrm{SD}}_{\mathrm{post}}}. \end{eqnarray*}

The correlation coefficient (r) reflects the test-retest reliability of the measure. As most studies did not report this coefficient directly, a moderate test-retest reliability level, widely accepted in the field, was assumed by setting *r* = 0.5. Furthermore, to assess the potential impact of this assumption on the pooled results, a sensitivity analysis was conducted by testing scenarios with *r* = 0.3 and *r* = 0.7.

For studies where pre- and pos*t*-test data were only available in figures, data extraction was performed using GetData Graph Digitizer software (version 2.26). If the full text or essential data were unavailable, the corresponding authors were contacted *via* email twice within one week to request the relevant information. The processes of literature screening and data extraction were performed independently by two investigators (ZX and SS). Any disagreements encountered during this process were resolved through consultation with a third investigator (ZX).

### Assessment of risk of bias

The methodological quality of all included studies was assessed using the Cochrane Risk of Bias tool within Review Manager 5.4 software. The quality assessment covered seven domains: random sequence generation, allocation concealment, blinding of participants and personnel, blinding of outcome assessment, incomplete outcome data, selective reporting, and other bias. Each domain included three evaluation criteria: “low risk of bias”, “unknown risk of bias” and “high risk of bias”. The study quality was ultimately assessed based on the number of “low risk of bias” domains, with the specific criteria being as follows: four or more domains rated as “low risk of bias”, two to three domains as ”unknown risk of bias”, and zero to one domain as ”high risk of bias”. This quality assessment was performed independently by the same two investigators, with any discrepancies resolved through consultation with a third investigator.

### Statistical analysis

Data analysis was conducted using Stata 15.0 software. The primary effect measure was the change value ± standard deviation before and after the intervention. For all outcome measures, the pooled effect size was analyzed using the standardized mean difference (SMD) with 95% confidence intervals. The *I*^2^ statistic was employed to assess heterogeneity among the studies: heterogeneity was considered negligible when *I*^2^ < 25%, moderate when 25% ≤*I*^2^ < 75%, and substantial when *I*^2^ ≥ 75% (Higgins et al., 2003). Due to the diversity of participants involved in sports science experiments and the variability of intervention methods, and to prevent the potential neglect of heterogeneity by fixed-effect models, all effect sizes in this study were analyzed using random-effects models (Chandler et al., 2019). In cases where significant heterogeneity was present, sensitivity (stepwise exclusion method) and subgroup analyses were conducted to explore potential sources of heterogeneity and to evaluate the robustness and reliability of the meta-analysis results. Subgroup analyses were conducted based on gender, intervention frequency, intervention duration, training equipment, and participants’ years of sport-specific training. To ensure sufficient statistical power and reliability, subgroup analyses were conducted only for outcomes with data available from 10 or more randomized controlled trial groups. A *p*-value of less than 0.05 was considered statistically significant.

Funnel plots were used to assess publication bias, and Egger’s test was further employed to quantify the results of publication bias. If publication bias was detected, the “trim-and-fill” method was used to adjust the funnel plot, and publication bias was reassessed after adjustment. To ensure the effectiveness of publication bias detection and the accuracy of the systematic review, publication bias was only assessed for meta-analyses that included more than 10 studies (Sterne et al., 2011).

## Results

### Study characteristics

This study ultimately included 15 articles involving 442 participants. The participants, aged between 14 and 25 years, were primarily basketball, soccer, and handball players. The intervention for the experimental group involved resistance training combined with elastic bands or chains, while the control group engaged in traditional resistance training or routine sport-specific training. The duration of the interventions in the included studies varied from 3 to 10 weeks, with a frequency of two to three times per week, and each session lasting 30 to 45 min (Table 1).

**Table 1:** Characteristics of study participants.

Studies	Genders	Sports	Sample size		Age		Experimental group				Control group	Key outcome indicators
			E	C	E	C	Interventions	Frequency	Duration		Interventions	
Tyagi et al. (2024)	Male	Basketball	15	15	24.00 ± 3.66	24.71 ± 3.83	VRT+ RT	3/week	45 min	8 weeks	FWT+ RT	VJ
Sawyer et al. (2021)	/	Football	20	20	/		VRT+ PST	3/week	/	3 weeks	FWT+ PST	VJ
Loturco et al. (2022)	Male	Football	12	13	18.50 ± 0.60		VRT+PST	3/week	/	4weeks	TRT+ PST	VJ, 5 m, 10 m
Katushabe & Kramer (2020)	Male	Football	9	8	20.47 ± 1.85		VRT+OST	3/week	/	6 weeks	TRT+ OST	VJ, RCOD
Aloui et al. (2019)	Male	Handball	15	15	18.30 ± 0.80	18.80 ± 0.80	VRT+ST	2/week	30 min	8 weeks	ST	5 m, 30 m, CMJ, SJ, T test, RCOD
Aloui et al. (2020)	Male	Handball	14	15	17.70 ± 0.30	18.10 ± 0.50	VRT+RT	2/week	/	8 weeks	RT	5 m, 30 m, CMJ, SJ, T test, RCOD
Gaamouri et al. (2023)	Female	Handball	17	17	15.70 ± 0.20	15.80 ± 0.20	VRT+RT	2/week	/	10 weeks	RT	CMJ, SJ, T test
Gaamouri et al. (2024)	Female	Handball	16	14	15.80 ± 0.30	15.80 ± 0.20	VRT+RT	2/week	30 min	10 weeks	RT	CMJ, SJ, T test
Hammami et al. (2022a)	Female	Handball	15	15	15.70 ± 0.30	15.70 ± 0.20	VRT+RT	2/week	30 min	10 weeks	RT	5 m, 10 m, 30 m, SJ, CMJ, T test, Illinois test
Hammami et al. (2022b)	Female	Handball	13	13	15.70 ± 0.20	15.80 ± 0.20	VRT+ST	2/week	/	10 weeks	ST	5 m, 10 m, 30 m, SJ, CMJ, T test, Illinois test
Hammami & Zmijewski (2024) (SEB)	Female	Handball	12	12	16.20 ± 0.40	16.30 ± 0.30	VRT+RT	2/week	30 min	10 weeks	RT	10 m, CMJ, SJ, Illinois test
Hammami & Zmijewski (2024) (CEB)	Female	Handball	12	12	16.20 ± 0.30	16.30 ± 0.30	VRT+RT	2/week	30 min	10 weeks	RT	10 m, CMJ, SJ, Illinois test
Raouf et al. (2022)	Male	Volleyball	14	13	14.86 ± 0.52	14.70 ± 0.36	VRT+ST	2/week	30 min	8 weeks	ST	CMJ
Jiang & Xu (2022) (10%)	Male	Basketball	11	11	15.73 ± 0.79	15.55 ± 0.93	VRT	2/week	/	6 weeks	85% 1RM squat training	VJ, 30 m
Jiang & Xu (2022) (20%)	Male	Basketball	11	11	15.18 ± 0.75	15.55 ± 0.93	VRT	2/week	/	6 weeks	85% 1RM squat training	VJ, 30 m
Jiang & Xu (2022) (30%)	Male	Basketball	11	11	15.45 ± 0.82	15.55 ± 0.93	VRT	2/week	/	6 weeks	85% 1RM squat training	VJ, 30 m
Izadi et al. (2019)	/	Badminton	12	12	20.72 ± 0.73	20.41 ± 0.79	VRT+RT	/	/	6 weeks	RT	VJ, 30 m
Izadi et al. (2019)	Male	Badminton	10	10	17.20 ± 0.70	17.30 ± 1.10	VRT+ST	2/week	/	8 weeks	ST	CMJ

**Notes.**

E experimental group C control group VRT variable resistance training FWT free weights training TRT traditional resistance training CMJ countermovement jump SJ squat Jump VJ vertical jump RCOD Repeated Change of Direction RT Regular training; PST pre-season training OST off season training ST season training

### Risk of bias in the included articles

**Figure 3:**
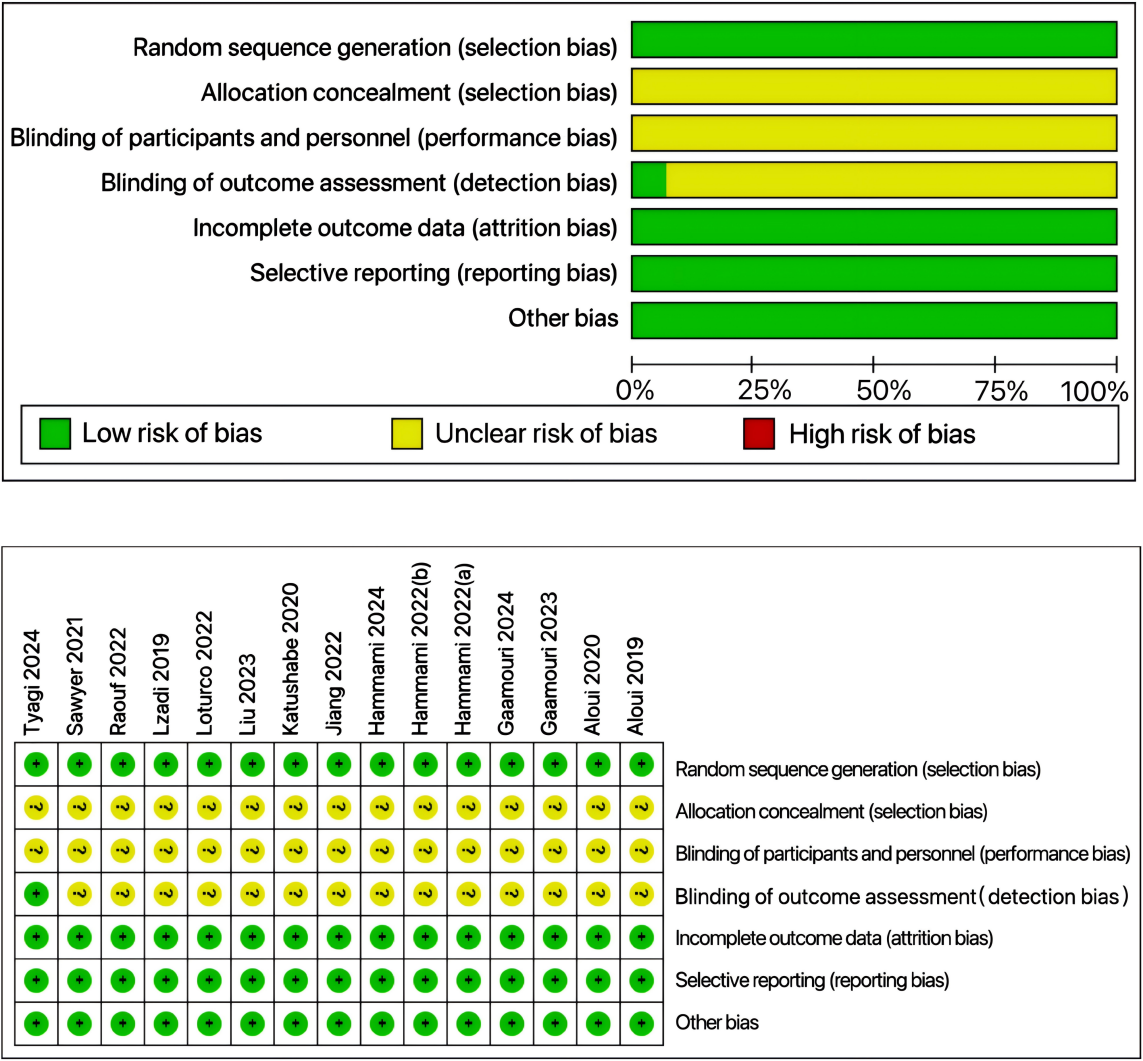
Risk of bias assessment chart. Studies: Aloui et al. (2020), Sawyer et al. (2021), Loturco et al. (2022), Hammami & Zmijewski (2024), Hammami et al. (2022a), Hammami et al. (2022b), Gaamouri et al. (2023), Gaamouri et al. (2024), Jiang & Xu (2022), Liu (2023), Izadi et al. (2019), Raouf et al. (2022), Tyagi et al. (2024).

The Cochrane Risk of Bias Assessment Tool was used in this study to evaluate the quality of the included studies. The results showed that all the included studies were randomized controlled trials, and one study implemented blinding of outcome assessors. Overall, all the included studies were rated as having a low risk of bias, indicating that the study results had high internal validity and reliability (Fig. 3).

### Meta-analysis results

### Jumping performance

**Figure 4:**
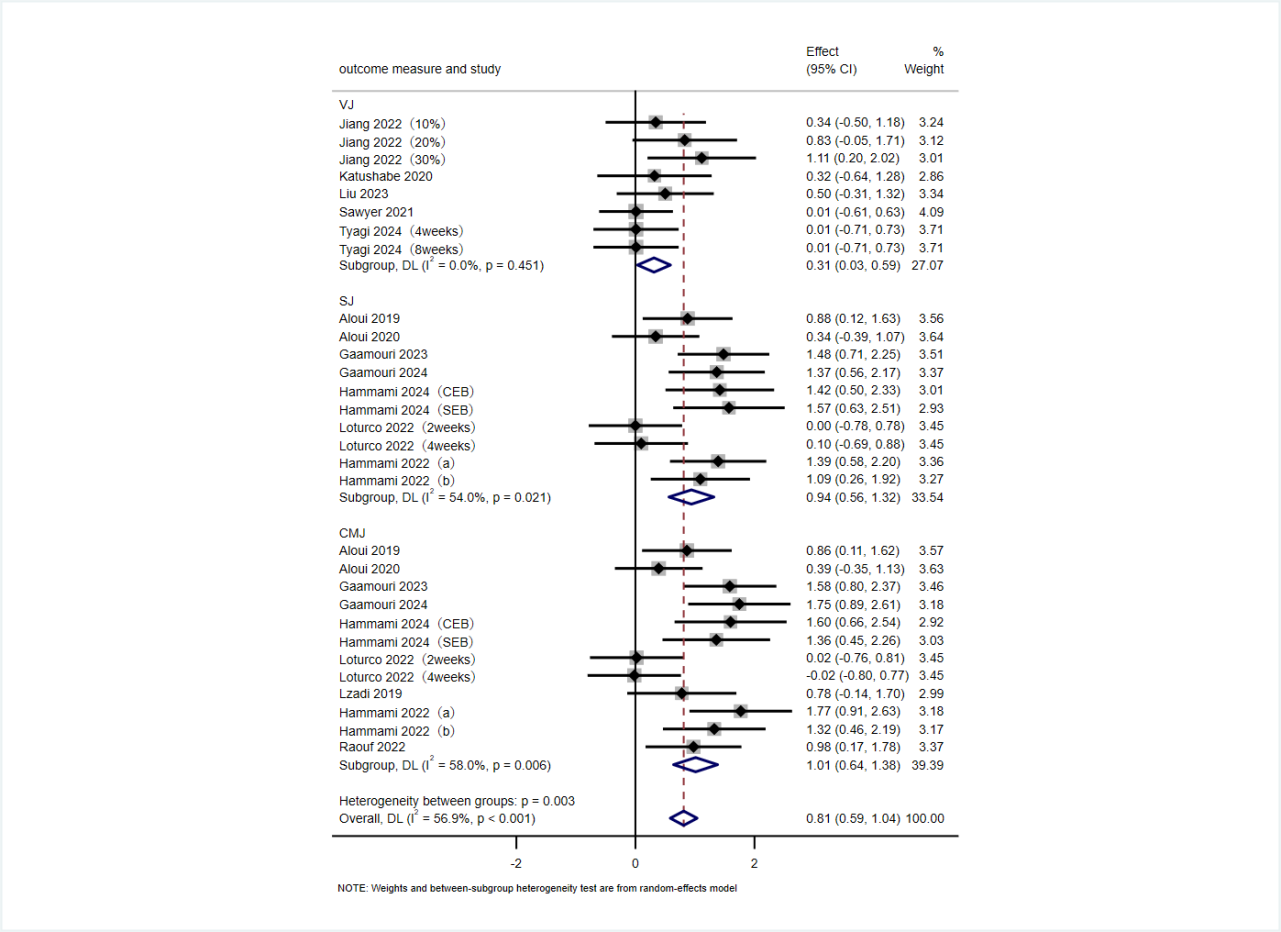
Forest plot of jumping performance. Studies: Aloui et al. (2020), Gaamouri et al. (2024), Loturco et al. (2022), Hammami & Zmijewski (2024), Hammami et al. (2022a), Hammami et al. (2022b), Gaamouri et al. (2023), Jiang & Xu (2022), Liu (2023), Izadi et al. (2019), Raouf et al. (2022), Tyagi et al. (2024).

A total of 15 articles, comprising 20 RCTs, utilized VJ, SJ and CMJ as outcome measures to evaluate the effects of VRT on athletes’ jumping performance. As shown in Fig. 4, in terms of the overall effect, VRT had a significant positive effect on improving athletes’ jumping performance (SMD = 0.81 cm, 95% CI [0.59 to 1.04], *p* < 0.001), with moderate heterogeneity among the studies (*I*^2^ = 56.9%, *p* < 0.001). The results of the subgroup analysis showed that VRT significantly improved athletes’ VJ (SMD = 0.31 cm, 95% CI [0.03 to 0.59], *p* = 0.027), SJ (SMD = 0.94 cm, 95% CI [0.56 to 1.32], *p* < 0.001), and CMJ (SMD = 1.01 cm, 95% CI [0.64 to 1.38], *p* < 0.001). The heterogeneity was negligible for VJ (*I*^2^ = 0.0%, *p* = 0.451), moderate for SJ (*I*^2^ = 54.0%, *p* = 0.021), and moderate for CMJ (*I*^2^ = 58.0%, *p* = 0.006).

Further subgroup analyses identified several factors contributing to the observed moderate heterogeneity, including sex (male, female), training frequency (2/week, 3/week), intervention duration (<6 weeks, 8 weeks, 10 weeks), intervention equipment (elastic bands, iron chain), and participants’ years of sport-specific training (<5 years, 5–7 years, >7 years) (Table 2).

**Table 2:** Results of subgroup analysis.

	N	Heterogeneity within subgroups	SMD within subgroups (95% CI)	Overall heterogeneity	Overall SMD (95% CI)
Jumping performance (gender)					
Male	16	*I*^2^ = 0.0%, *p* = 0.467	0.41 (0.21, 0.60), *p* = 0.000	*I*^2^ = 55.4%, *p* < 0.001	0.86 (0.62, 1.09), *p* = 0.000
Female	12	*I*^2^ = 0.0%, *p* = 0.998	1.47 (1.23, 1.72), *p* = 0.000		
Jumping performance (frequency)					
2/weeks	21	*I*^2^ = 14.0%, *p* = 0.276	1.13 (0.93, 1.32), *p* = 0.000	*I*^2^ = 58.1%, *p* < 0.001	0.82 (0.59, 1.06), *p* = 0.000
3/weeks	8	*I*^2^ = 0.0%, *p* = 0.100	0.04 (−0.23, 0.31), *p* = 0.765		
Jumping performance (weeks)					
<6 weeks	10	*I*^2^ = 0.0%, *p* = 0.594	0.27 (0.01, 0.52), *p* = 0.038	*I*^2^ = 56.9%, *p* < 0.001	0.81 (0.59, 1.04), *p* = 0.000
=8 weeks	8	*I*^2^ = 5.6%, *p* = 0.387	0.50 (0.22, 0.78), *p* = 0.000		
=10 weeks	12	*I*^2^ = 0.0%, *p* = 0.998	1.47 (1.23, 1.72), *p* = 0.000		
Jumping performance (equipment)					
Elastic bands	27	*I*^2^ = 62.0%, *p* < 0.001	0.82 (0.57, 1.08), *p* = 0.000	*I*^2^ = 56.9%, *p* < 0.001	0.81 (0.59, 1.04), *p* = 0.000
Iron chain	4	*I*^2^ = 0.0%, *p* = 0.671	0.75 (0.31, 1.19), *p* = 0.001		
Jumping performance (specialized training history)					
<5 years	7	*I*^2^ = 0.0%, *p* = 0.464	0.39 (0.08, 0.70), *p* = 0.014	*I*^2^ = 57.7%, *p* < 0.001	0.81 (0.57, 1.06), *p* = 0.000
5–7 years	14	*I*^2^ = 67.1%, *p* < 0.001	0.90 (0.52, 1.28), *p* = 0.000		
>7 years	5	*I*^2^ = 0.0%, *p* = 0.409	1.12 (0.76, 1.48), *p* = 0.000		
Sprint performance (gender)					
Male	7	*I*^2^ = 59.5%, *p* = 0.022	−0.73 (−1.21, −0.25), *p* = 0.003	*I*^2^ = 75.2%, *p* < 0.001	−1.20 (−1.65, −0.75), *p* = 0.000
Female	8	*I*^2^ = 78.3%, *p* < 0.001	−1.67 (−2.36, −0.98), *p* = 0.000		
Sprint performance (weeks)					
<6 weeks	4	*I*^2^ = 0.0%, *p* = 1.000	−0.04 (−0.45, −0.37), *p* = 0.852	*I*^2^ = 75.9%, *p* < 0.001	−1.13 (−1.57, −0.69), *p* = 0.000
=8 weeks	4	*I*^2^ = 0.0%, *p* = 0.805	−1.22 (−1.62, −0.83), *p* = 0.000		
=10 weeks	8	*I*^2^ = 78.3%, *p* < 0.001	−1.67 (−2.36, −0.98), *p* = 0.000		
Sprint performance (equipment)					
Elastic bands	13	*I*^2^ = 72.2%, *p* < 0.001	−1.38 (−1.83, −0.92), *p* = 0.000	*I*^2^ = 75.9%, *p* < 0.001	−1.13 (−1.57, −0.69), *p* = 0.000
Iron chain	3	*I*^2^ = 0.0%, *p* = 0.994	−0.04 (−0.52, −0.45), *p* = 0.880		
Sprint performance (specialized training history)					
<5 years	4	*I*^2^ = 0.0%, *p* = 0.100	−0.04 (−0.45, −0.37), *p* = 0.852	*I*^2^ = 76.0%, *p* < 0.001	−1.10 (−1.59, −0.62), *p* = 0.000
5–7 years	4	*I*^2^ = 0.0%, *p* = 0.839	−1.38 (−1.81, −0.95), *p* = 0.000		
>7 years	5	*I*^2^ = 78.0%, *p* = 0.001	−1.77 (−2.60, −0.93), *p* = 0.000		
Change of direction (gender)					
Male	5	*I*^2^ = 6.1%, *p* = 0.372	−1.04 (−1.42, −0.66), *p* = 0.000	*I*^2^ = 70.6%, *p* < 0.001	−1.66 (−2.12, −1.19), *p* = 0.000
Female	8	*I*^2^ = 71.3%, *p* < 0.001	−2.12 (−2.76, −1.48), *p* = 0.000		
Change of direction (specialized training history)					
<6 years	3	*I*^2^ = 84.4%, *p* = 0.002	−1.57 (−2.94, −0.19), *p* = 0.026	*I*^2^ = 74.1%, *p* < 0.001	−1.77 (−2.32, −1.23), *p* = 0.000
6–7 years	6	*I*^2^ = 48.1%, *p* = 0.086	−1.50 (−1.99, −1.00), *p* = 0.000		
>7 years	2	*I*^2^ = 88.8%, *p* = 0.003	−3.00 (−5.41, −0.58), *p* = 0.015		

#### Sprint performance

**Figure 5:**
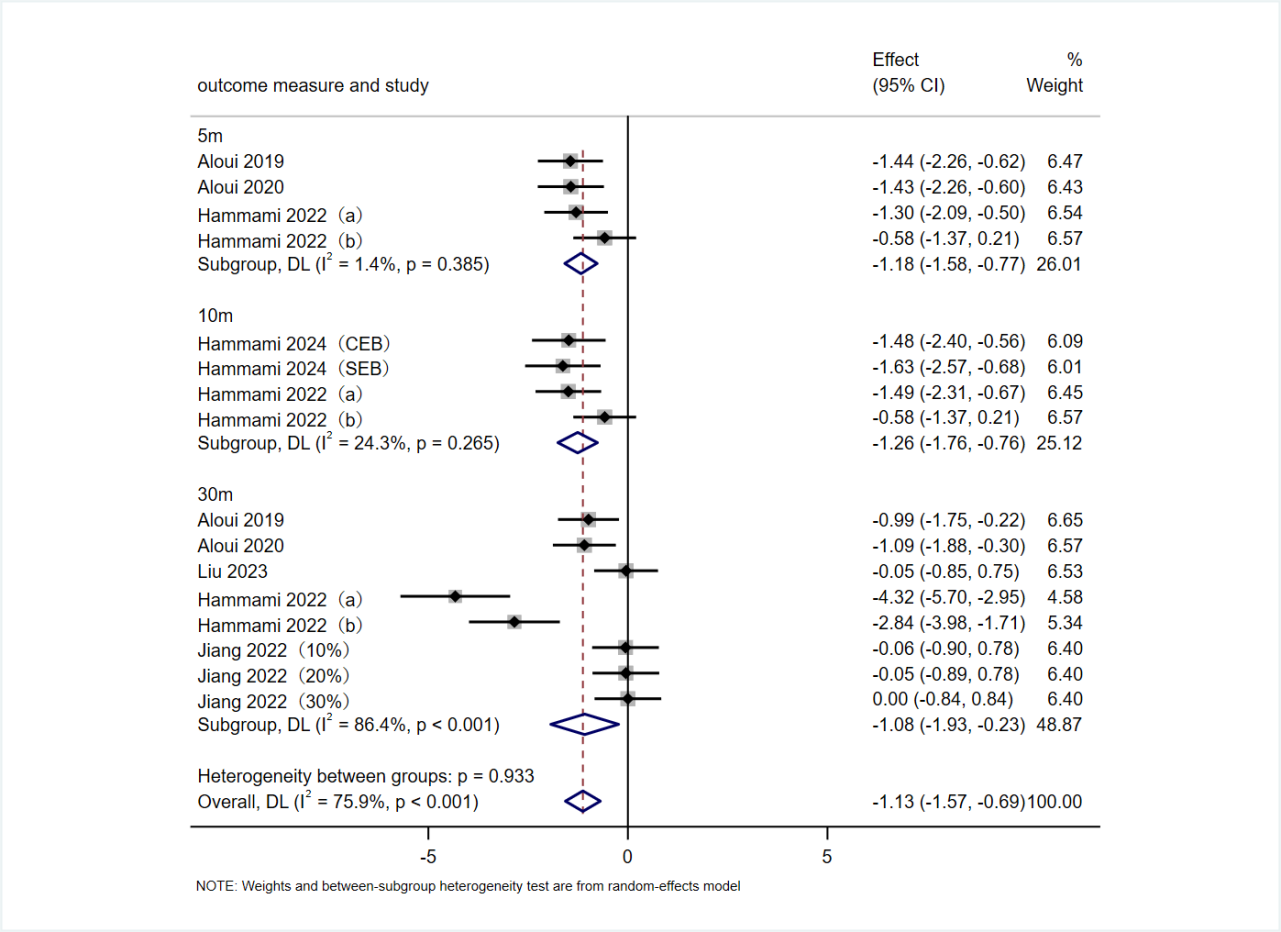
Forest plot of sprinting performance. Studies: Aloui et al. (2020), Gaamouri et al. (2024), Jiang & Xu (2022), Liu (2023), Izadi et al. (2019), Raouf et al. (2022), Tyagi et al. (2024), Loturco et al. (2022), Hammami & Zmijewski, (2024), Hammami et al., (2022a), Hammami et al. (2022b), Gaamouri et al. (2023), Aloui et al. (2019).

A total of seven articles, comprising 10 RCTs, utilized 5-m, 10-m, and 30-m sprint tests as outcome measures to evaluate the effects of VRT on sprint performance in athletes. As shown in Fig. 5, in terms of the overall effect, VRT had a significant positive effect on improving athletes’ sprinting performance (SMD = −1.13 s, 95% CI [−1.57 to −0.69], *p* < 0.001), with substantial heterogeneity among the studies (*I*^2^ = 75.9%, *p* < 0.001). The results of the subgroup analysis showed that VRT significantly improved athletes’ 5-m sprint (SMD = −1.18 s, 95% CI [−1.58 to −0.77], *p* < 0.001), 10-m sprint (SMD = −1.26 s, 95% CI [−1.76 to −0.76], *p* < 0.001), and 30-m sprint (SMD = −1.08 s, 95% CI [−1.93 to −0.23], *p* = 0.013). Heterogeneity was negligible for 5-m sprint (*I*^2^ = 1.4%, *p* = 0.385) and 10-m sprint (*I*^2^ = 24.3%, *p* = 0.265), but high for 30-m sprint (*I*^2^ = 86.4%, *p* < 0.001).

As presented in Table 2, subgroup analyses identified several factors contributing to the observed high heterogeneity, including sex (male, female), intervention duration (<6 weeks, 8 weeks, 10 weeks), intervention equipment (elastic bands, iron chain), and participants’ years of sport-specific training (<5 years, 5–7 years, >7 years).

#### Change of direction performance

**Figure 6:**
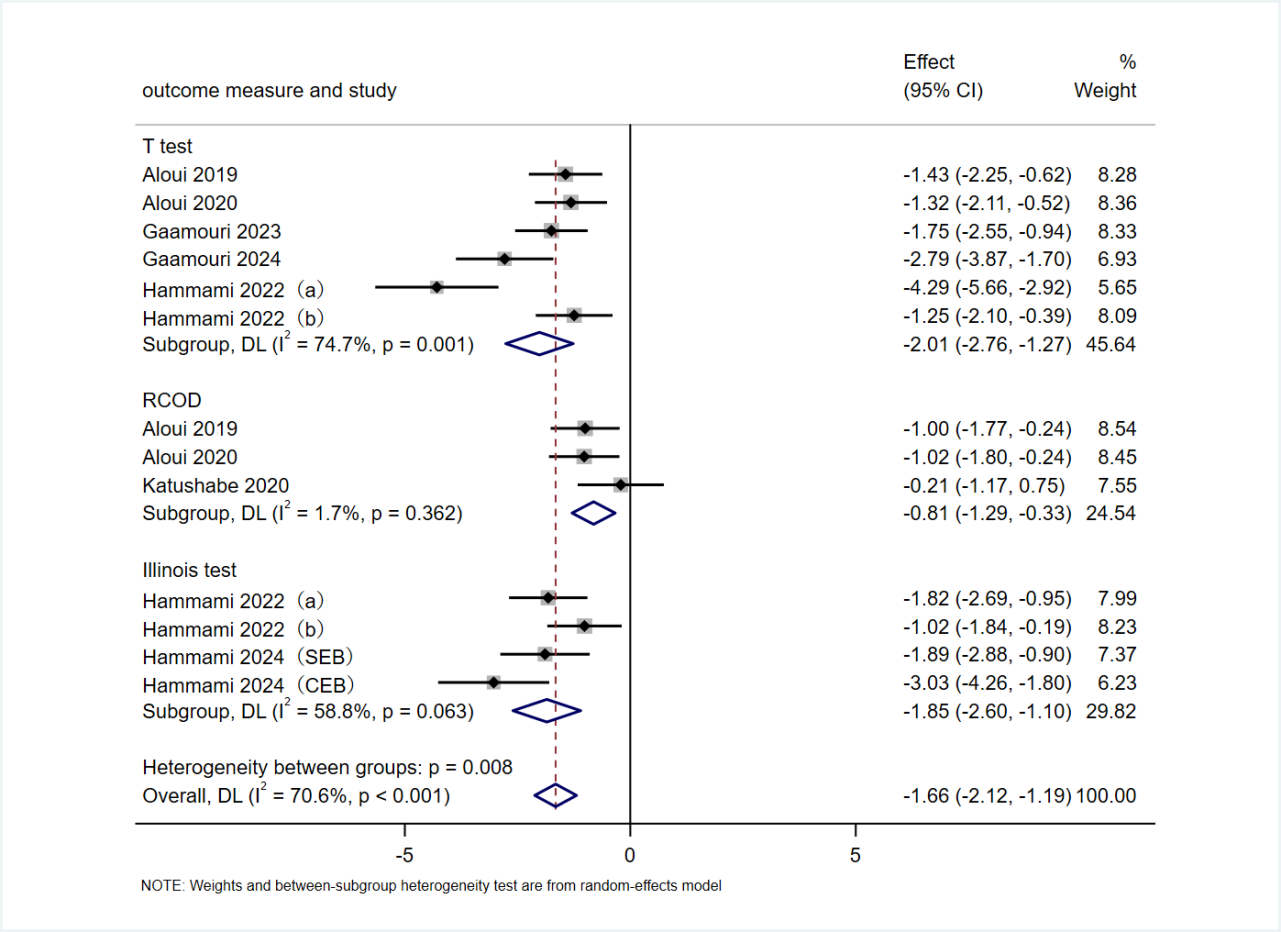
Forest plot of COD performance. Studies: Aloui et al. (2020), Gaamouri et al. (2024), Jiang & Xu (2022), Liu (2023), Izadi et al. (2019), Raouf et al. (2022), Tyagi et al. (2024), Aloui et al. (2019), Hammami & Zmijewski (2024), Hammami et al. (2022a), Hammami et al. (2022b), Gaamouri et al. (2023).

A total of eight articles, comprising nine RCTs, utilized T test, RCOD test, and Illinois test as outcome measures to evaluate the effects of VRT on change-of-direction performance in athletes. As shown in Fig. 6, the overall effect indicated that VRT had a significant positive impact on athletes’ COD performance (SMD = −1.66 s, 95% CI [−2.12 to −1.19], *p* < 0.001). The studies exhibited moderate heterogeneity (*I*^2^ = 70.6%, *p* < 0.001). The subgroup analysis results showed that VRT significantly improved performance in the T test (SMD = −2.01 s, 95% CI [−2.76 to −1.27], *p* < 0.001), RCOD (SMD = −0.81 s, 95% CI [−1.29 to −0.33], *p* = 0.001), and Illinois test (SMD = −1.85 s, 95% CI [−2.60 to −1.10], *p* < 0.001). Moderate heterogeneity was observed for the T test (*I*^2^ = 74.7%, *p* = 0.001) and Illinois test (*I*^2^ = 58.8%, *p* = 0.063), while heterogeneity was negligible for RCOD (*I*^2^ = 1.7%, *p* = 0.362).

As shown in Table 2, subgroup analyses identified sex (male, female) and participants’ years of sport-specific training (<6 years, 6–7 years, >7 years) as factors contributing to the observed moderate heterogeneity.

### Risk of bias assessment

**Figure 7:**
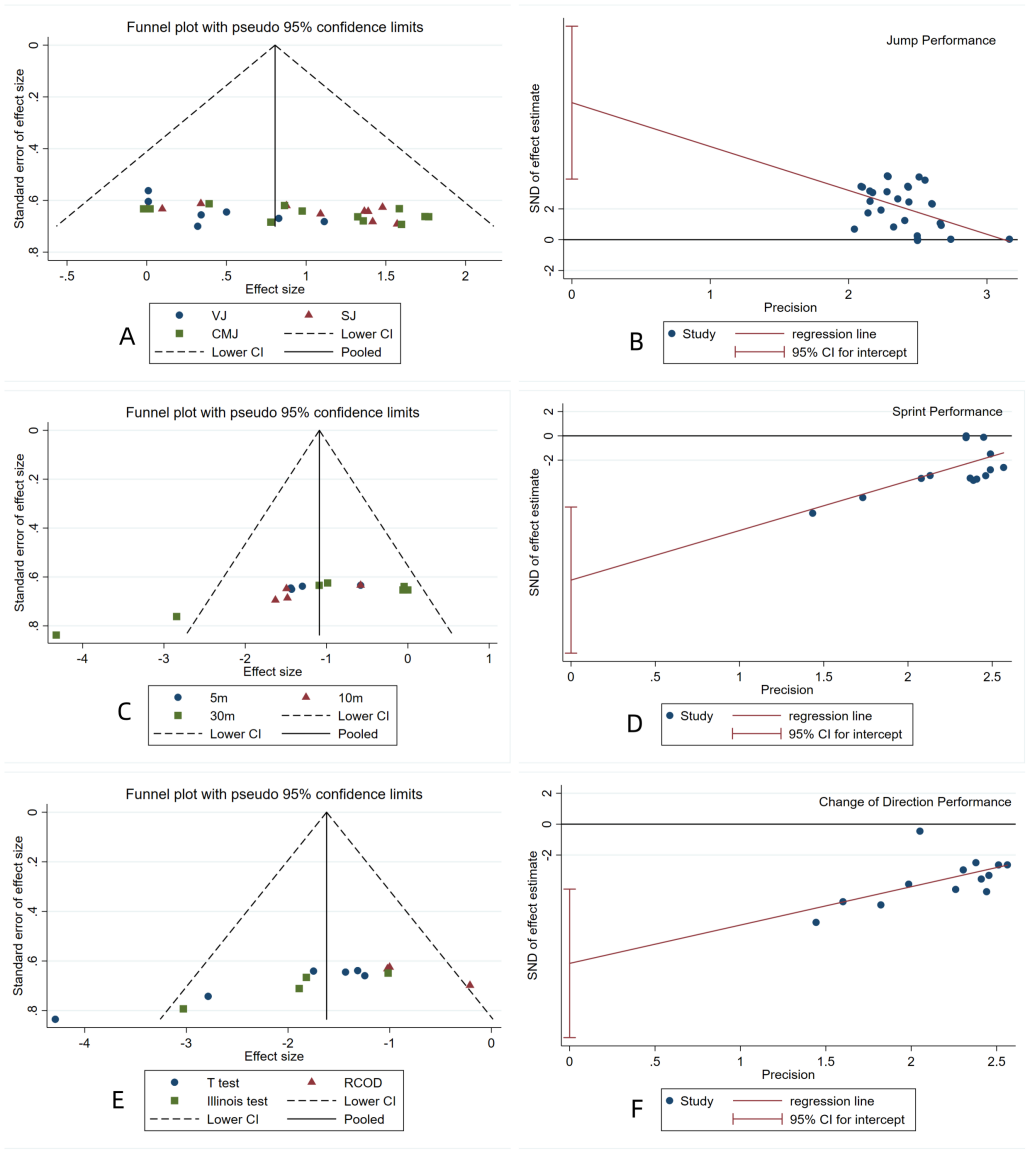
(A–F) Funnel plots and Egger’s test plots.

**Figure 8:**
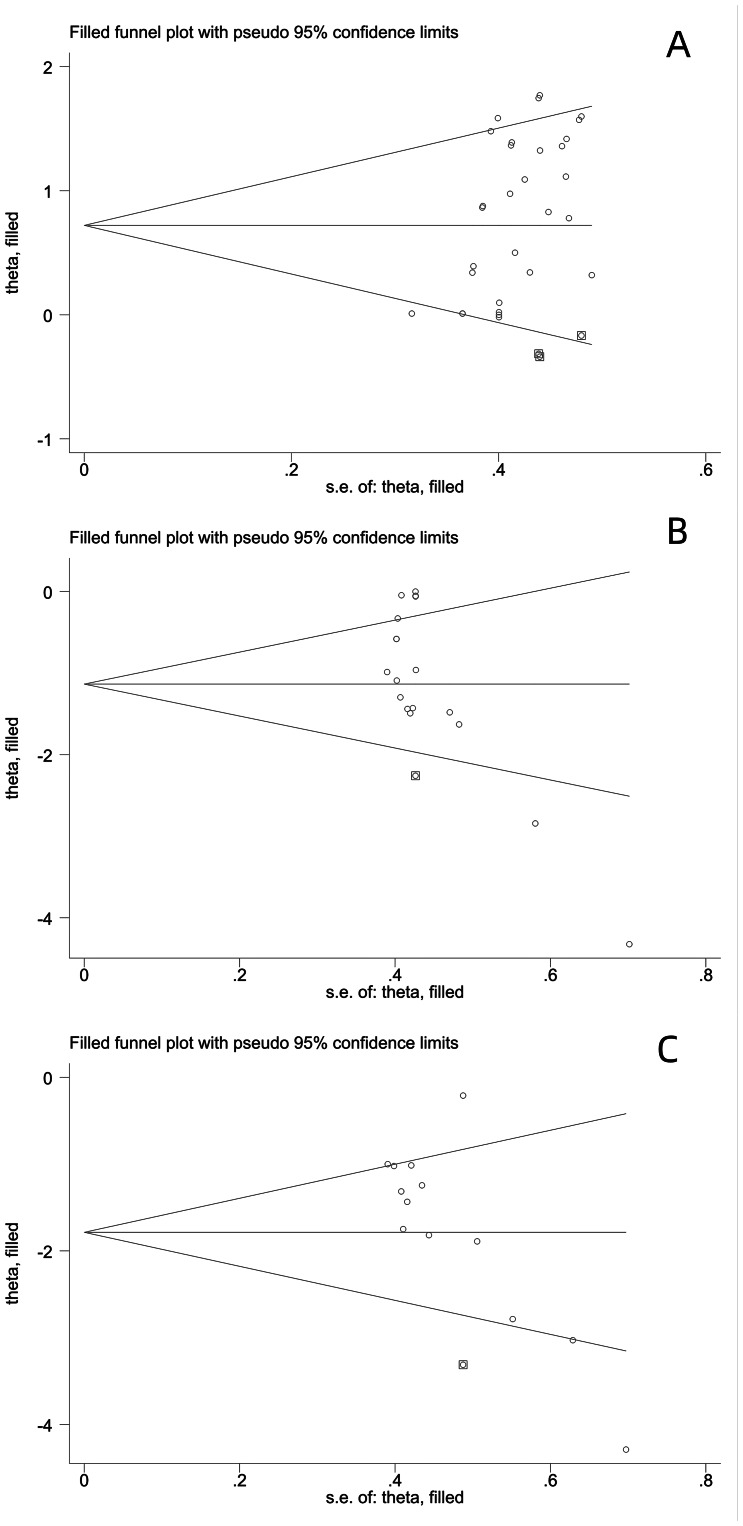
(A–C) Funnel plot of jumping, sprinting and COD performance after trim and fill.

As shown in Fig. 7, the funnel plot was visually inspected and revealed an asymmetrical distribution of effect sizes, suggesting the presence of publication bias. The results of Egger’s test indicated that publication bias existed in jumping performance (*t* = 3.68, *p* = 0.001), sprinting performance (*t* = −4.26, *p* = 0.001), and COD performance (*t* = −4.11, *p* = 0.002) (Fig. 7). Further analysis using the “trim-and-fill” method showed that for jumping performance, three studies were missing (*Q* = 67.274, *p* = 0.000), and the pooled effect size using the random-effects model was 0.811 (95% CI [0.585 to 1.036]); for sprinting performance, one study was missing (*Q* = 62.334, *p* = 0.000), and the pooled effect size was −1.128 (95% CI [−1.566 to −0.690]); for COD performance, one study was missing (*Q* = 40.767, *p* = 0.000), and the pooled effect size was −1.655 (95% CI [−2.119 to −1.192]). After filling in the missing studies, the meta-analysis results for jumping performance were *Q* = 82.300, *p* = 0.000, with a pooled effect size of 0.721 (95% CI [0.492 to 0.949]); for sprinting performance, *Q* = 72.493, *p* = 0.000, with a pooled effect size of −1.210 (95% CI [−1.653 to −0.767]); for COD performance, *Q* = 53.434, *p* = 0.000, with a pooled effect size of −1.786 (95% CI [−2.278 to −1.294]). After filling in the missing studies, the funnel plot no longer showed significant asymmetry, and the results did not reverse, indicating that the combined results were robust (Fig. 8).

### Sensitivity analysis

**Figure 9:**
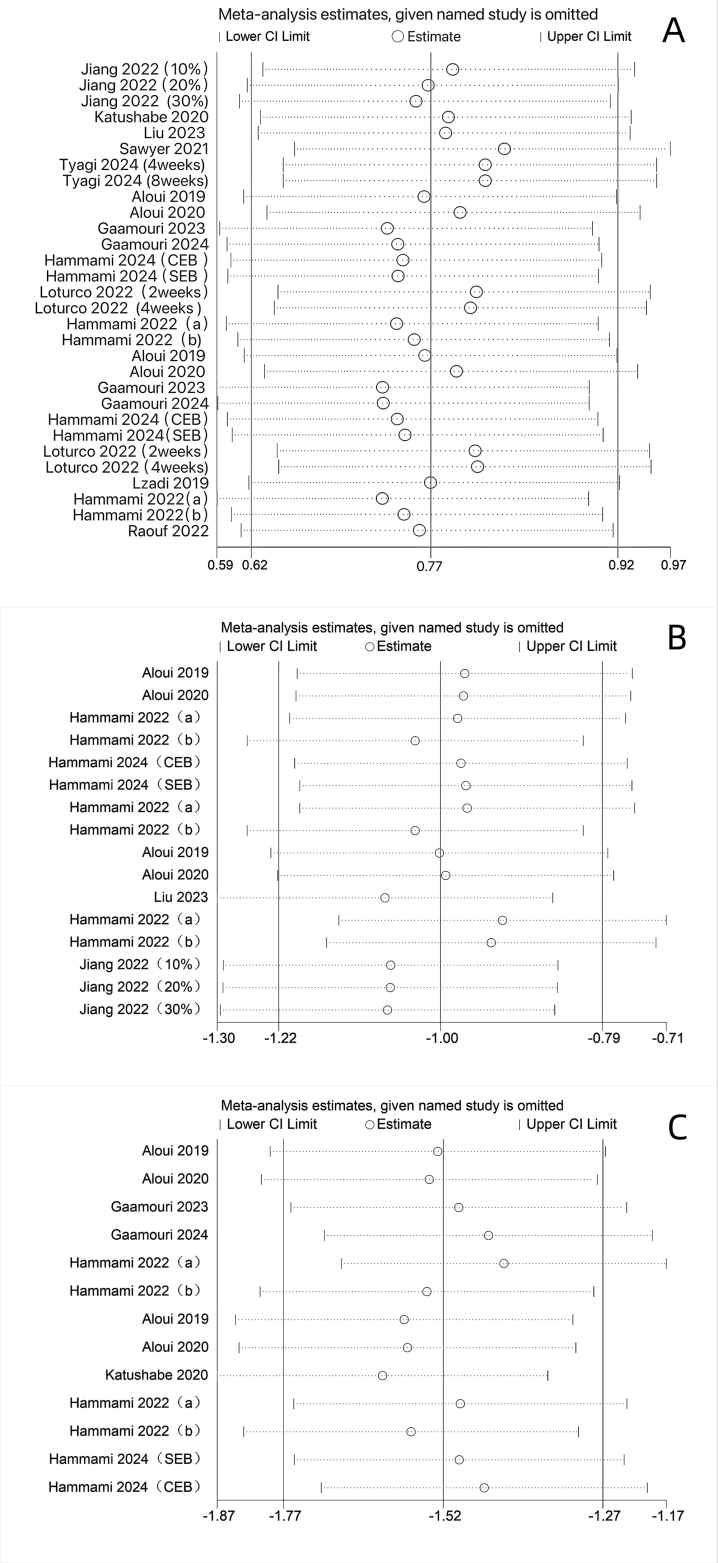
(A–C) Sensitivity analysis of jumping, sprinting and COD performance. Studies: Aloui et al. (2020), Gaamouri et al. (2024), Jiang & Xu (2022), Liu (2023), Izadi et al. (2019), Raouf et al. (2022), Tyagi et al. (2024), Hammami et al. (2022a), Hammami et al. (2022b), Aloui et al. (2019), Katushabe & Kramer (2020).

Sensitivity analysis was conducted to assess the potential impact of each included study on the meta-analysis results. As shown in Fig. 9, the pooled results fluctuated within a range of 0.77 for jump performance, −1.00 for sprint performance, and −1.52 for change-of-direction performance, indicating relative stability of the overall findings. To examine the influence of the r on the results, we performed additional analyses using different r values. When r was set to 0.3, the results for jump, sprint, and change-of-direction performance fluctuated around 0.69, −0.95, and −1.43, respectively. When r was set to 0.7, the corresponding results were approximately 1.04, −1.32, and −2.07, respectively (File S6). Although the numerical values varied with different r values, the direction of effects remained consistent across all scenarios, with no reversal of results observed. This further supports the robustness of our findings.

## Discussion

### Jumping performance

The study results demonstrate that VRT may be an effective method for enhancing athletes’ jumping performance (VJ, *p* = 0.027; SJ, *p* < 0.001; CMJ, *p* < 0.001). However, despite extensive research investigating the effects of VRT on jump performance, considerable inconsistencies persist across studies. To clarify these discrepancies, this study employed, for the first time, a meta-analytic approach to quantitatively synthesize previous research, aiming to provide a comprehensive effect estimate and a more holistic evaluation of the overall impact of VRT on jumping ability. Further analysis suggests that variations in training protocols and participant characteristics are likely key factors contributing to the heterogeneous outcomes. For instance, Andersen et al. (2018) and Katushabe & Kramer (2020) reported positive effects of VRT. The former study observed improvements in both arm-supported and unsupported CMJ performance among handball players following a 9-week elastic band strength training program. These results may be closely associated with the relatively long intervention duration, suggesting that sustained training can lead to more substantial enhancements (Andersen et al., 2018). The latter study also indicated that the significant improvement in VJ performance after VRT was primarily attributable to the generation of greater FRD (Katushabe & Kramer, 2020). However, other studies have reached divergent conclusions. Aloui et al. (2019) found no significant differences in VJ and CMJ performance between subjects who underwent 8 weeks of VRT training and those who engaged in Conventional Resistance Training (CRT). This lack of significant improvement may be attributed to the subjects’ already high level of physical fitness, which suggests that their jumping ability had reached a plateau. Consequently, greater training intensity or higher training volume might be necessary to further enhance their jumping performance (Aloui et al., 2019).

Jumping ability is a critical component in many sports and is a core element in the training regimens of numerous athletes. Particularly in ball games and track and field events, exceptional jumping ability not only provides athletes with an aerial advantage during competitions but also enhances explosive power and coordination, thereby improving overall athletic performance (Kollias, Panoutsakopoulos & Papaiakovou, 2004). Moreover, good jumping ability can reduce impact forces during landing, thereby lowering the risk of injury (Rössler et al., 2014). VRT offers unique advantages in improving athletes’ jumping performance. First, VRT can significantly enhance muscle activation and the recruitment of motor units. During VRT, as the limbs extend, the external moment arm of specific joints gradually decreases, while the skeletal muscle lever system gains greater mechanical advantage (McMaster, Cronin & McGuigan, 2009). This allows for greater force production during limb extension. In this context, adding extra external load at the end range of the movement may further increase the number of activated motor units and raise the firing frequency of motor neurons that innervate fast-twitch muscle fibers (Andersen et al., 2016). Second, VRT can alter muscle recruitment patterns. By providing a continuously changing load throughout the joint range of motion, VRT enables muscles to operate closer to their maximum capacity. Athletes’ adaptation to external loads can lead to greater recruitment of Type IIX fibers (Andersen et al., 2020; Anderson, Sforzo & Sigg, 2008). These fibers have higher contraction speeds and force output capabilities, and their increased recruitment during jumping helps athletes rapidly generate force and improve jumping performance (Haff & Triplett, 2016). Moreover, VRT can effectively enhance the eccentric control ability of lower limb muscles. The execution of jumping movements largely depends on the stretch-shortening cycle (SSC) of muscles (Li, Yu & Yang, 2019). During landing, the hip, knee, and ankle joints must quickly decelerate body mass to cushion the impact. In the early phase of the eccentric stage, VRT reduces the load to allow higher eccentric velocities, and then increases resistance during the concentric phase. This forces athletes to call upon stronger neural drive during the transition from deceleration to acceleration (Fuentes-García, Malchrowicz-Mosko & Castañeda Babarro, 2024). This training method improves the efficiency of force conversion during jumping, thereby increasing jump height and distance.

It should be noted that the present study is subject to a certain degree of publication bias, suggesting that studies with non-significant results may have been underreported, potentially leading to an overestimation of the observed effect sizes. While sensitivity analysis confirmed the robustness of our findings, moderate heterogeneity was still observed in the overall effects. Subgroup analyses indicated that sex, training frequency, intervention duration, training equipment, and participants’ years of sport-specific training were potential sources of heterogeneity. Regarding participant characteristics, sex differences may be attributed to greater adaptive potential in baseline strength among female participants (Roberts, Nuckols & Krieger, 2020). The influence of training years reflects that athletes with longer training backgrounds generally possess superior foundational strength and more stabilized motor patterns (Suchomel et al., 2018). In terms of training protocols, the impact of intervention duration was supported by Sawyer et al. (2021), who found that a 3-week VRT intervention failed to induce significant improvements in VJ performance. In contrast, most studies reporting positive outcomes implemented interventions lasting no less than 6 weeks, suggesting that the training effects of VRT may require longer accumulation periods to manifest (Sawyer et al., 2021). Furthermore, analysis of training frequency revealed that a twice-weekly schedule produced superior effects compared to a thrice-weekly regimen. This may be explained by the high-intensity, neurologically dominant nature of VRT, which places substantial demands on central nervous system recovery and musculotendinous adaptation. A lower training frequency likely facilitates more complete supercompensation, thereby enhancing the quality of individual sessions and optimizing long-term training adaptations.

### Sprinting performance

The study results demonstrate that VRT may be an effective method for enhancing athletes’ sprinting performance (5-m, *p* < 0.001; 10-m, *p* < 0.001; 30-m, *p* = 0.013). CRT primarily enhances sprinting ability by increasing muscle strength and power output, which translates directly into greater propulsion during the start and acceleration phases of sprinting (Van Hooren, Aagaard & Blazevich, 2024). However, VRT has shown a greater advantage in improving sprint performance compared to CRT. Hammami & Zmijewski (2024) confirmed the efficacy of VRT in enhancing sprint performance through a 10-week training intervention, attributing the improvements to enhanced neuromuscular adaptations (Hammami & Zmijewski, 2024). This perspective is further supported and elaborated by Wyland, Van Dorin & Reyes (2015), whose research found that VRT, as an activation method, enables athletes to generate and sustain greater force for a longer duration during the concentric phase of strength training (Wyland, Van Dorin & Reyes, 2015). These studies collectively reveal the effectiveness of VRT in enhancing sprint performance.

However, these results should be interpreted with caution due to the presence of publication bias and high heterogeneity identified in this review. Through subgroup analysis, this study identified gender, intervention duration, intervention equipment, and participants’ specific training experience as potential sources of heterogeneity. The patterns of influence for gender, intervention duration, and training experience on the results were similar to those observed for jump performance. Furthermore, differences in training equipment may also affect training outcomes. The primary equipment used for VRT includes chains and elastic bands; however, due to their distinct kinetic characteristics, these two types of equipment induce different loading profiles within the concentric movement range, potentially leading to divergent training effects (Arandjelović, 2010; Gonzalo-Skok et al., 2017). Some inconsistencies were noted among the studies included in this review. For instance, the study by Loturco et al. (2022) found that while VRT improved maximal strength and jump performance, sprint performance decreased in mid-term testing (Loturco et al., 2022). This seemingly paradoxical finding might be partially explained by Wong et al. (2010), who suggested that concurrent engagement in substantial sport-specific routine practice of an aerobic nature might impede the development of anaerobic sprint capacity (Wong et al., 2010). Additionally, substantial heterogeneity was particularly evident for the 30-meter sprint outcome. The studies by Hammami et al. (2022a) and Hammami et al. (2022b) were identified as major contributors to this heterogeneity, as both reported more pronounced improvements in 30-meter sprint performance. It is hypothesized that their training protocols were more effective in activating the knee extensor and flexor muscles, while the variable resistance provided by elastic bands further enhanced the power output of the involved muscle groups, thereby translating more effectively into improved sprint speed (Hammami et al., 2022a; Hammami et al., 2022b).

Sprinting is a movement that requires rapid contraction and sustained force output of the lower limb muscles and is an extremely critical quality for athletes in competitive sports (Haugen et al., 2019). In team sports such as rugby and handball, superior sprinting performance enables athletes to quickly break through defensive lines, chase opponents, seize advantageous positions, or complete key offensive and defensive actions. In sprint events, sprinting performance is directly tested and often determines success. The mechanisms by which VRT improves sprint performance can be explained from multiple aspects. VRT can optimize neuromuscular adaptations and overcome sticking points. Studies have found that compared to CRT, the variable resistance provided by elastic bands or chains requires muscles to dynamically adjust their contraction patterns throughout the entire movement, matching the biomechanical characteristics of the human body. This allows muscles to approach their maximum contraction levels across the full range of motion (Griffin & Cafarelli, 2005; Hammami et al., 2022a). This not only promotes the synchronization of motor unit recruitment and enhances neuromuscular coordination (Hammami & Zmijewski, 2024), but also effectively translates muscle power output into improved sprint performance (Janusevicius et al., 2017).VRT can also effectively enhance sprint performance by improving the rate of RFD and the SSC. During the concentric contraction phase, VRT provides progressive resistance, enabling muscles to continuously generate higher forces as the mechanical advantage of joint angles improves. At the same time, it shortens the SSC time to promote the rapid release of elastic potential energy (Findley, 2004; Rhea, Kenn & Dermody, 2009). Moreover, during sprinting, the lower limb muscles need to coordinate contraction and relaxation in a short period of time to achieve rapid stride frequency and length. VRT changes muscle recruitment patterns and working methods, allowing muscles to be effectively trained at different joint angles and improving muscle coordination and control (Andersen et al., 2022). This helps athletes better control their body posture and movement techniques during sprinting, thereby enhancing sprint efficiency and performance.

### Change of direction

The study results demonstrate that VRT may be an effective method for enhancing athletes’ COD performance (T-test: *p* < 0.001; RCOD: *p* = 0.001; Illinois test: *p* < 0.001). Compared to the body of research on jumping and sprint performance, studies investigating the effects of VRT on COD ability remain relatively limited. Therefore, this review provides comprehensive data support for this field from an integrative perspective. Overall, the results of this study align with the majority of existing studies on the impact of VRT on COD performance, which suggest that VRT may be a superior method to CRT for improving athletes’ COD performance (Gaamouri et al., 2023; Hammami et al., 2022a). For example, Hammami et al. (2022a) and Hammami et al. (2022b) found that after VRT intervention, athletes’ T test and Illinois test scores significantly improved. The primary reason is that during VRT, greater force can be generated in the latter half of the concentric phase and the first half of the eccentric phase of each repetition, while the transition efficiency between the concentric and eccentric phases is also enhanced. This training method effectively increases hamstring strength, providing substantial support for improved COD performance (Hammami et al., 2022a; Lopes et al., 2019).

However, the presence of publication bias necessitates cautious interpretation of these results. Additionally, moderate heterogeneity further limits the generalizability of the findings. Subgroup analysis identified gender and specific training experience as significant sources of heterogeneity, with female participants demonstrating superior training effects compared to males, and athletes with longer training histories exhibiting more pronounced improvements. This suggests that training background and physiological characteristics moderate the response to VRT. Notably, some inconsistencies exist between our aggregated results and certain literature. For example, our overall findings diverge from those of Andersen et al. (2018) and Katushabe & Kramer (2020), who reported no significant improvements in COD performance following VRT interventions (Andersen et al., 2018; Sheppard et al., 2006). Beyond the potential influence of intervention duration, this discrepancy might be explained by the possibility that COD relies more heavily on coordinative abilities rather than solely on pure strength or neuromuscular adaptations (Andersen et al., 2018; Sheppard et al., 2006). Consequently, to effectively enhance COD ability, incorporating agility-based exercises such as multi-directional movements into resistance training programs may be necessary to achieve simultaneous improvements in both strength and COD performance.

COD is a comprehensive reflection of multiple athletic qualities, including strength, speed, agility, and coordination, and is equally crucial for athletes. In sports such as basketball, football, and rugby, which require rapid movement and flexible directional adjustments, COD is often a key factor in determining competitive performance (Brughelli et al., 2008). The mechanisms by which VRT significantly improves athletes’ jumping and sprinting performance also apply to the enhancement of COD ability. In addition to these mechanisms, the improvement of COD performance by VRT is mainly based on the following two unique reasons: First, the enhancement of strength and power output is a key factor in improving COD ability. The essence of COD lies in the ability to rapidly shift the body’s center of mass while maintaining stability (Dos’ Santos et al., 2020). When an athlete’s strength is increased, the stability and support capacity of the lower limb muscles are significantly enhanced, thereby effectively reducing deceleration or loss of balance due to instability of the center of mass (Dos’ Santos et al., 2017). Moreover, increased power output typically implies an increase in Type II muscle fibers. This increase enables athletes to rapidly generate force during the moment of COD, maintaining the normal execution of the movement. Therefore, the improvement of individual strength and power output is a primary mechanism for enhanced COD performance (Sheppard & Young, 2006). Second, the improvement of proprioception and balance ability also plays an important role in enhancing COD ability. During COD, athletes need to accurately perceive changes in body position and center of mass, and quickly adjust body posture and muscle state based on these changes (Brughelli et al., 2008). The characteristic of VRT is that it provides constantly changing external resistance for athletes. This variability forces athletes to continuously adjust during training to cope with changes in resistance (Lin et al., 2022). As a result, athletes need to engage more core muscles to maintain balance, while stimulating the adaptation of muscle spindles and Golgi tendon organs—two types of receptors within the muscles—to the changing resistance. With increased training duration, the sensitivity and accuracy of muscle spindles and Golgi tendon organs will also improve, thereby further enhancing athletes’ proprioception and balance ability during COD.

## Conclusions

Based on the synthesis of current evidence, this study demonstrates that VRT exerts positive effects on athletes’ jumping performance (VJ, SJ, CMJ), sprint performance (5 m, 10 m, 30 m), and change-of-direction performance (*T*-test, RCOD, Illinois test). However, due to the heterogeneity among included studies and potential publication bias, these conclusions should be interpreted with caution. Overall, the existing evidence supports VRT as a potentially effective method for enhancing lower-limb explosive power in athletes, though its definitive efficacy warrants further validation through additional high-quality research.

### Limitations and prospects

This study systematically explored the impact of VRT on athletes’ lower limb explosive power. However, it is undeniable that this study also has some limitations. First, the age of the participants included in the analysis are mainly around 20 years old, and they are mostly adolescent athletes, which limits the applicability of the study conclusions to a broader group of athletes. Second, the study results show considerable heterogeneity, which may affect our comprehensive and accurate understanding of the training effects of VRT.

Based on the findings and limitations of the study, future research should conduct experimental studies targeting adult athletes or investigate the training effects of VRT at different stages of adolescence in adolescent athletes to gain a more comprehensive and in-depth understanding of the impact of VRT on athletes’ lower limb explosive power. In addition, considering the differences in physical fitness and neuromuscular function between males and females, exploring the different effects of VRT on muscle strength and explosive power in males and females will be of great significance.

## Supplemental Information

10.7717/peerj.20644/supp-1 Supplemental Information 1 Inclusion and Exclusion of the Literature

10.7717/peerj.20644/supp-2 Supplemental Information 2 Search strategy

10.7717/peerj.20644/supp-3 Supplemental Information 3 PRISMA checklist

10.7717/peerj.20644/supp-4 Supplemental Information 4 Target audience

10.7717/peerj.20644/supp-5 Supplemental Information 5 Raw data

10.7717/peerj.20644/supp-6 Supplemental Information 6 Results of the Sensitivity Analysis Using Different r-values

## Additional Information and Declarations

### Data Availability

The following information was supplied regarding data availability: This is a systematic review/meta-analysis.
