# Supplementary material for: Effects of variable resistance training on lower limb explosive power in athletes: a systematic review and meta-analysis
Source: PeerJ. 2026 Feb 4;14:e20644. doi: 10.7717/peerj.20644 (PMC12882733; doi:10.7717/peerj.20644)
Supplement: Supplemental Information 2 [file peerj-14-20644-s002.docx]

| Database | Web of Science | |
| --- | --- | --- |
| Search Date | March 23, 2025 | |
| Search Period | From the inception of database to March 23, 2025 | |
| No. | Search strategy | Literatures retrieved |
| #1 | AB=("elastic band" OR "rubber band" OR "theraband" OR "elastic tubing" OR "chain" OR "variable resistance" OR "accommodating resistance" OR "elastic band" OR "elastic tubing chain" OR "variable resistance training" OR "VRT" OR "chain training" OR "elastic training" OR "elastic resistance" OR "variable cam" OR "bands resistance" OR "elastic tube" OR "rubber tube" OR "pneumatic machine" OR "pneumatic resistance" OR "chain resistance" OR "chains free weight") | 1,179,069 |
| #2 | AB=("power" OR "strength" OR "force" OR "speed" OR "jump" OR "height" OR "velocity" OR "distance" OR "output" OR "horizontal jump" OR "vertical jump" OR "SJ" OR "CMJ" OR "explosive strength" OR "lower limbs power" OR "explosive force" OR "explosive effort" OR "explosive power" OR "force of explosion" OR "bursting force" OR "explosive strength" OR "standing long jump" OR "SLJ" OR "DJ" OR "VJ" OR "10m" OR "20m" OR "30m" OR "Countermovement jump" OR "squat jump" OR "drop jump" OR "sprint performance" OR "50m" OR "explosiveness" OR "Change of direction" OR "T test" OR "illinois" OR "change direction" OR "sensitive quality" OR "agility" OR "directional change" OR "COD" OR "Repeated Change of Direction") | 8,161,707 |
| #3 | AB=("Athletes" OR "Professional Athletes" OR "Elite Athletes" OR "player" OR "Professional Athletes" OR "Elite Athletes" OR "College Athletes" OR "player") | 136,662 |
| #4 | #3 AND #2 AND #1 | 589 |

| Database | PubMed | |
| --- | --- | --- |
| Search Date | March 23, 2025 | |
| Search Period | From the inception of database to March 23, 2025 | |
| No. | Search strategy | Literatures retrieved |
| #1 | ("elastic band"[Title/Abstract]) OR ("rubber band"[Title/Abstract]) OR ("theraband"[Title/Abstract]) OR ("elastic tubing"[Title/Abstract]) OR ("chain"[Title/Abstract]) OR ("variable resistance"[Title/Abstract]) OR ("accommodating resistance"[Title/Abstract]) OR ("elastic band"[Title/Abstract]) OR ("elastic tubing chain"[Title/Abstract]) OR ("variable resistance training"[Title/Abstract]) OR ("chain training"[Title/Abstract]) OR ("elastic training"[Title/Abstract]) OR ("elastic resistance"[Title/Abstract]) OR ("variable cam"[Title/Abstract]) OR ("bands resistance"[Title/Abstract]) OR ("elastic tube"[Title/Abstract]) OR ("rubber tube"[Title/Abstract]) OR ("pneumatic machine"[Title/Abstract]) OR ("pneumatic resistance"[Title/Abstract]) OR ("chain resistance"[Title/Abstract]) OR ("chains free weight"[Title/Abstract]) | 769,518 |
| #2 | ("power"[Title/Abstract]) OR ("strength"[Title/Abstract])OR ("force"[Title/Abstract]) OR ("speed"[Title/Abstract]) OR ("jump"[Title/Abstract]) OR ("height"[Title/Abstract]) OR ("velocity"[Title/Abstract]) OR ("output"[Title/Abstract]) OR ("horizontal jump"[Title/Abstract]) OR ("vertical jump"[Title/Abstract]) OR ("SJ"[Title/Abstract]) OR ("CMJ"[Title/Abstract]) OR ("explosive strength"[Title/Abstract]) OR ("lower limbs power"[Title/Abstract]) OR ("explosive force"[Title/Abstract]) OR ("explosive effort"[Title/Abstract]) OR ("explosive power"[Title/Abstract]) OR ("force of explosion"[Title/Abstract]) OR ("bursting force"[Title/Abstract]) OR ("explosive strength"[Title/Abstract]) OR ("standing long jump"[Title/Abstract]) OR ("SLJ"[Title/Abstract]) OR ("DJ"[Title/Abstract]) OR ("VJ"[Title/Abstract]) OR ("10m"[Title/Abstract]) OR ("20m"[Title/Abstract]) OR ("30m"[Title/Abstract]) OR ("Countermovement jump"[Title/Abstract]) OR ("squat jump"[Title/Abstract]) OR ("drop jump"[Title/Abstract]) OR ("sprint performance"[Title/Abstract]) OR ("50m"[Title/Abstract]) OR ("explosiveness"[Title/Abstract] OR "Change of direction"[Title/Abstract] OR "T test"[Title/Abstract] OR "illinois"[Title/Abstract] OR "change direction"[Title/Abstract] OR "sensitive quality"[Title/Abstract] OR "agility"[Title/Abstract] OR "directional change"[Title/Abstract] OR "COD"[Title/Abstract] OR "Repeated Change of Direction"[Title/Abstract]) | 2,074,560 |
| #3 | ("Athletes"[Title/Abstract]) OR ("Professional Athletes"[Title/Abstract]) OR ("Elite Athlete"[Title/Abstract]) OR ("player"[Title/Abstract]) OR ("Professional Athletes"[Title/Abstract]) OR ("Elite Athletes"[Title/Abstract]) OR ("College Athletes"[Title/Abstract]) OR ("player"[Title/Abstract]) | 99,341 |
| #4 | #3 AND #2 AND #1 | 475 |

| Database | Scopus | |
| --- | --- | --- |
| Search Date | March 23, 2025 | |
| Search Period | From the inception of database to March 23, 2025 | |
| No. | Search strategy | Literatures retrieved |
| #1 | TITLE-ABS("elastic band" OR "rubber band" OR "theraband" OR "elastic tubing" OR "chain" OR "variable resistance" OR "accommodating resistance" OR "elastic band" OR "elastic tubing chain" OR "variable resistance training" OR "VRT" OR "chain training" OR "elastic training" OR "elastic resistance" OR "variable cam" OR "bands resistance" OR "elastic tube" OR "rubber tube" OR "pneumatic machine" OR "pneumatic resistance" OR "chain resistance" OR "chains free weight") | 1,867,306 |
| #2 | TITLE-ABS("power" OR "strength" OR "force" OR "speed" OR "jump" OR "height" OR "velocity" OR "output" OR "horizontal jump" OR "vertical jump" OR "SJ" OR "CMJ" OR "explosive strength" OR "lower limbs power" OR "explosive force" OR "explosive effort" OR "explosive power" OR "force of explosion" OR "bursting force" OR "explosive strength" OR "standing long jump" OR "SLJ" OR "DJ" OR "VJ" OR "10m" OR "20m" OR "30m" OR "Countermovement jump" OR "squat jump" OR "drop jump" OR "sprint performance" OR "50m" OR "explosiveness" OR "Change of direction" OR "T test" OR "illinois" OR "change direction" OR "sensitive quality" OR "agility" OR "directional change" OR "COD" OR "Repeated Change of Direction") | 12,664,273 |
| #3 | TITLE-ABS("Athletes" OR "Professional Athletes" OR "Elite Athletes" OR "player" OR "Professional Athletes" OR "Elite Athletes" OR "College Athletes" OR "player") | 356,558 |
| #4 | #3 AND #2 AND #1 | 1800 |

| Database | ProQuest | |
| --- | --- | --- |
| Search Date | March 23, 2025 | |
| Search Period | From the inception of database to March 23, 2025 | |
| No. | Search strategy | Literatures retrieved |
| #1 | ABSTRACT("elastic band" OR "rubber band" OR "theraband" OR "elastic tubing" OR "chain" OR "variable resistance" OR "accommodating resistance" OR "elastic tubing chain" OR "variable resistance training" OR "VRT" OR "chain training" OR "elastic training" OR "elastic resistance" OR "variable cam" OR "bands resistance" OR "elastic tube" OR "rubber tube" OR "pneumatic machine" OR "pneumatic resistance" OR "chain resistance" OR "chains free weight") | 781,736 |
| #2 | ABSTRACT("power" OR "strength" OR "force" OR "speed" OR "jump" OR "height" OR "velocity" OR "output" OR "horizontal jump" OR "vertical jump" OR "SJ" OR "CMJ" OR "explosive strength" OR "lower limbs power" OR "explosive force" OR "explosive effort" OR "explosive power" OR "force of explosion" OR "bursting force" OR "standing long jump" OR "SLJ" OR "DJ" OR "VJ" OR "10m" OR "20m" OR "30m" OR "Countermovement jump" OR "squat jump" OR "drop jump" OR "sprint performance" OR "50m" OR "explosiveness" "Change of direction" OR "T test" OR "illinois" OR "change direction" OR "sensitive quality" OR "agility" OR "directional change" OR "COD" OR "Repeated Change of Direction") | 3,581,032 |
| #3 | ABSTRACT("Athletes" OR "Professional Athletes" OR "Elite Athletes" OR "player" OR "Professional Athletes" OR "Elite Athletes" OR "College Athletes" OR "player") | 430,855 |
| #4 | #3 AND #2 AND #1 | 630 |
