# Supplementary material for: Effects of variable resistance training on lower limb explosive power in athletes: a systematic review and meta-analysis
Source: PeerJ. 2026 Feb 4;14:e20644. doi: 10.7717/peerj.20644 (PMC12882733; doi:10.7717/peerj.20644)
Supplement: Supplemental Information 4 [file peerj-14-20644-s004.docx]

Our paper, "Effects of Variable Resistance Training on Lower Limb Explosive Power in Athletes: A Systematic Review and Meta-Analysis," targets the following primary audiences:

**1.Sports Science Researchers**

Researchers in the field of sports science are one of the main audiences for this paper. They are interested in the mechanisms and effects of training methods, particularly how different training approaches can enhance athletic performance. This study provides a systematic summary and data support regarding the effects of Variable Resistance Training (VRT) on lower limb explosive power, which can serve as a foundation and direction for future research.

**2. Coaches and Training Experts**

Professional sports coaches and training experts are another important audience for this paper. Coaches need to continuously update and optimize training methods to enhance athlete performance. This study provides evidence of the effectiveness of VRT, helping coaches design more scientifically sound training programs, especially in relation to explosive power training, to better meet the needs of athletes.

**3. Athletes**

Athletes engaged in sports that require explosive power (such as basketball, soccer, athletics, etc.) are also a target audience for this research. By understanding the role of VRT in enhancing explosive power, athletes can adjust their training methods to improve their competitive performance and training outcomes.

**4. Sports Medicine and Rehabilitation Professionals**

Professionals in sports medicine and rehabilitation (such as physiotherapists and athletic trainers) are also likely to be interested in this research. Understanding the application and effects of VRT can assist them in designing training programs that ensure athletes enhance their abilities safely during injury prevention and recovery.
