# Supplementary material for: Effects of variable resistance training on lower limb explosive power in athletes: a systematic review and meta-analysis
Source: PeerJ. 2026 Feb 4;14:e20644. doi: 10.7717/peerj.20644 (PMC12882733; doi:10.7717/peerj.20644)
Supplement: Supplemental Information 6 [file peerj-14-20644-s006.docx]

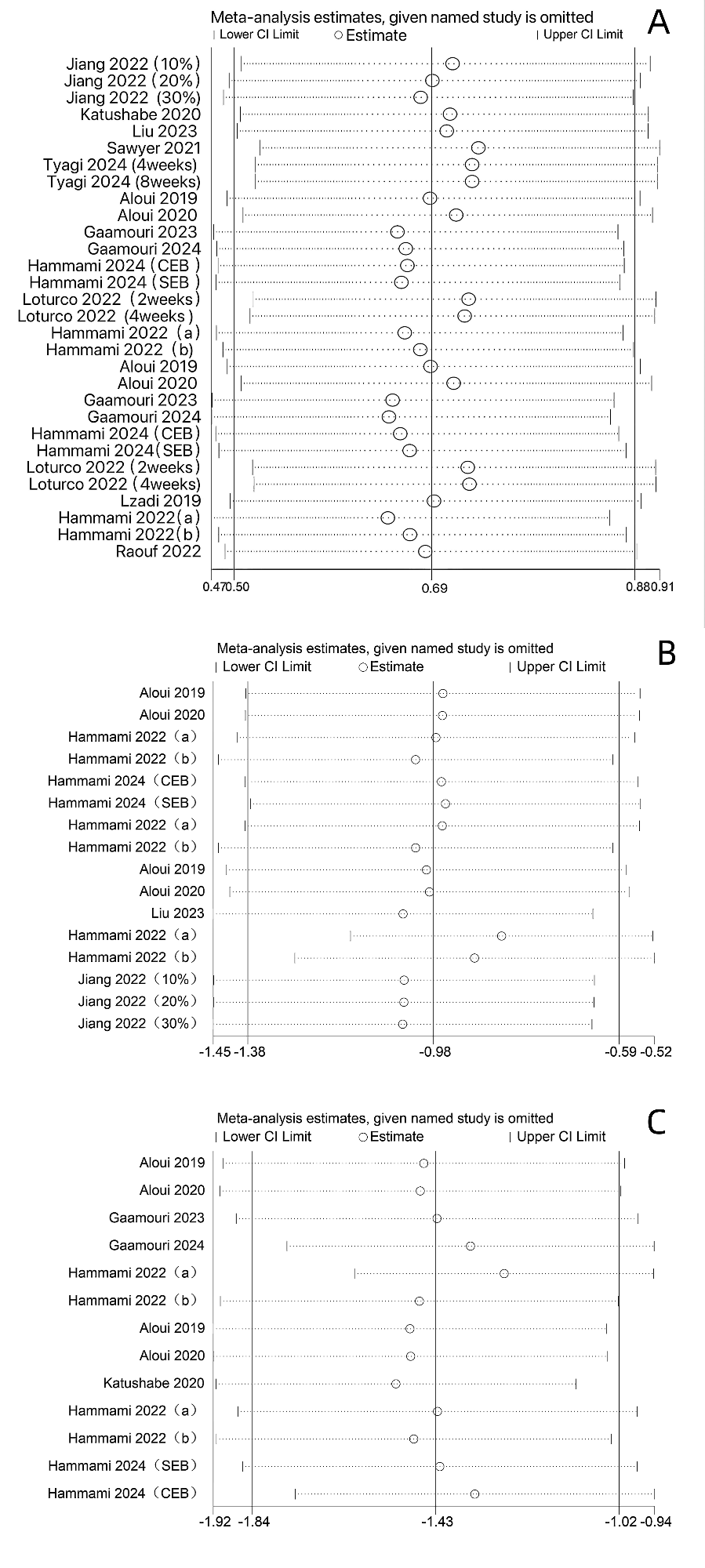


Figure1 Sensitivity Analysis with r = 0.3 (A: jumping performance; B: sprinting performance; C: change-of-direction performance)


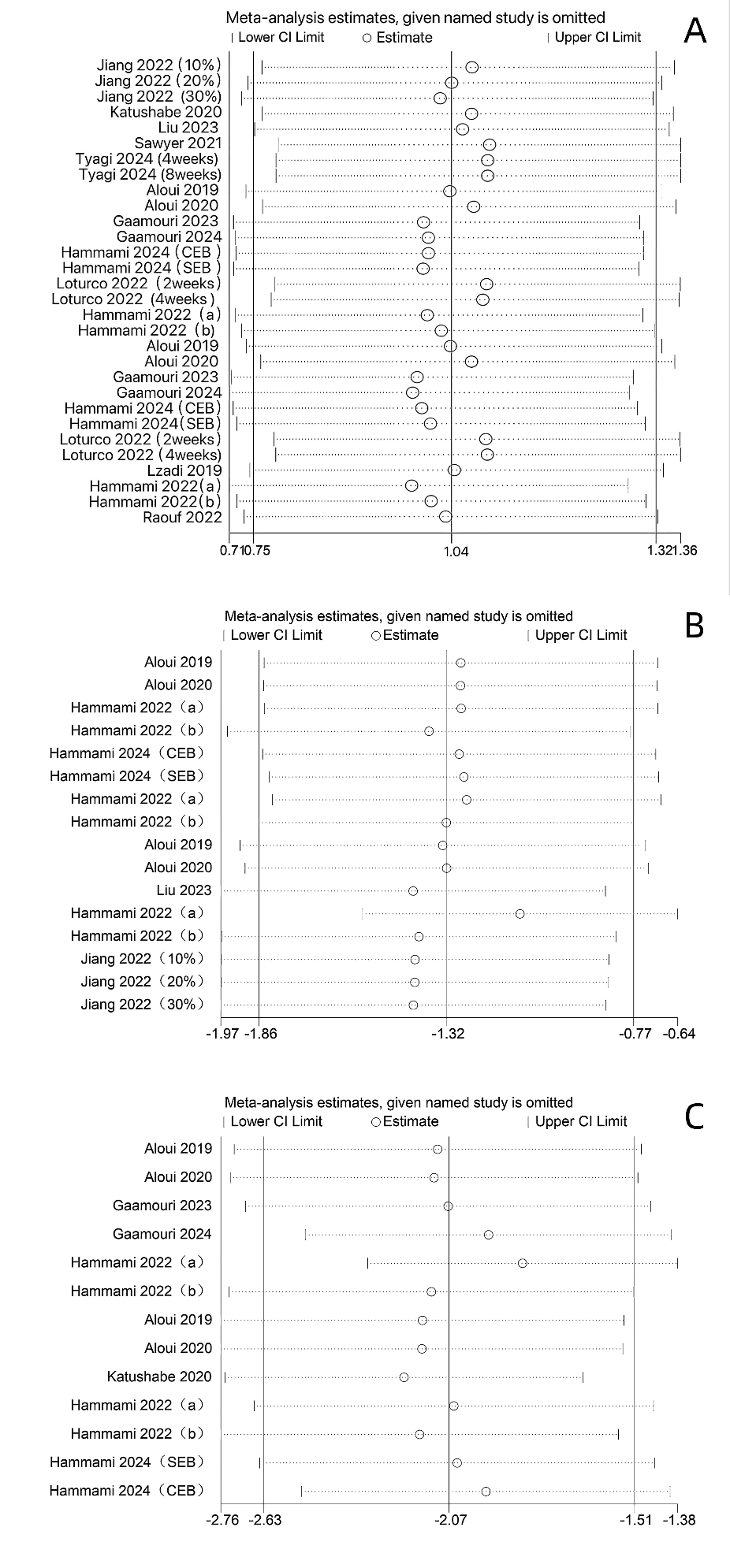


Figure2 Sensitivity Analysis with r = 0.7 (A: jumping performance; B: sprinting performance; C: change-of-direction performance)
